# Biochemical analysis of the diverse functions of the HTLV-1-encoded oncoprotein Tax

**DOI:** 10.1186/1742-4690-11-S1-O53

**Published:** 2014-01-07

**Authors:** Jennifer K Nyborg, Dinaida Egan, Neelam Sharma

**Affiliations:** 1 Department of Biochemistry and Molecular Biology, Colorado State University, Fort Collins, CO, USA

## 

The human T-cell leukemia/lymphoma virus, type I (HTLV-I)-encoded Tax protein is a potent transcription factor and oncoprotein. Therefore, studies on Tax function have provided critical insights into fundamental mechanisms of transcriptional activation and clues to the process of malignant transformation. Biochemical analysis of recombinant, purified Tax has yielded significant insights into many of its diverse functions, despite the absence of potentially important post-translational modifications. We will present evidence demonstrating the potent transcriptional activation function of Tax in vitro, including dissection of the interactions between Tax and specific cellular transcription factors that led to the discovery of Tax-mediated disruptions in chromatin architecture. This presentation will also describe several important findings regarding the molecular defect in Tax M47, which carries point mutations in the activation domain that render the Tax mutant defective for HTLV transcription. Comparison of the physical and functional properties of wild-type and mutant Tax reveals similar profiles in size-exclusion chromatography (SEC) and nearly identical affinity profiles in activator/co activator complex formation; paradoxical observations given the pronounced defect in the ability of Tax M47 to activate HTLV-1 transcription. Importantly, proteolysis assays revealed that TaxM47 produces a distinct digestion pattern relative to wild-type Tax, suggesting that M47 folds into a distinct tertiary structure that is not detected in SEC analysis. Together, these findings further underscore the outstanding potential of native and recombinant Tax to probe the molecular basis of transcriptional activation in higher eukaryotes, as well as the significant potential of Tax in uncovering diverse, yet fundamental, cellular processes.

